# The Role of Heparan Sulfate in CCL26-Induced Eosinophil Chemotaxis

**DOI:** 10.3390/ijms23126519

**Published:** 2022-06-10

**Authors:** Alexandra Pum, Maria Ennemoser, Tanja Gerlza, Andreas J. Kungl

**Affiliations:** 1 Institute of Pharmaceutical Sciences, Department of Pharmaceutical Chemistry, Karl-Franzens-University Graz, Schubertstrasse 1, 8010 Graz, Austria; alexandra_pum@gmx.at (A.P.); maria.ennemoser@uni-graz.at (M.E.); tanja.gerlza@uni-graz.at (T.G.); 2 Antagonis Biotherapeutics GmbH, Strasserhofweg 77a, 8045 Graz, Austria

**Keywords:** CCL26, eotaxin-3, glycosaminoglycans, GAG, heparan sulfate, heparinase C, transendothelial cell migration, transmigration, eosinophils, GAG-binding site, trans-theory, alanine scan, site-directed mutagenesis

## Abstract

Proinflammatory chemokine ligand 26 (CCL26, eotaxin-3) mediates transendothelial cell migration of eosinophils by binding and activating the G-protein-coupled (GPC) chemokine receptor 3 on the surface of eosinophilic cells. Here we have investigated the role of glycosaminoglycans (GAGs) as potential co-receptors in the process of CCL26-induced eosinophil chemotaxis. For this purpose, we have first identified the GAG-binding site of CCL26 by a site-directed mutagenesis approach in the form of an alanine screening. A panel of GAG-binding-deficient mutants has been designed, generated, and analyzed with respect to their binding affinities to heparan sulphate (HS) by isothermal fluorescence titration studies. This showed that basic amino acids in the α-helical part of CCL26 are strongly involved in GAG-binding. In chemotaxis experiments, we found that decreased GAG-binding affinity correlated with decreased chemotactic activity, which indicates an involvement of GAGs in eosinophil migration. This was further proven by the negative impact of heparinase III treatment and, independently, by the incubation of eosinophils with an anti heparan sulfate antibody. We finally investigated eosinophils’ proteoglycan (PG) expression patterns by real-time PCR, which revealed the highest expression level for serglycin. Including an anti-serglycin antibody in CCL26-induced eosinophil migration experiments reduced the chemotaxis of these immune cells, thereby proving the dependence of eosinophil mobilization on the proteoglycan serglycin.

## 1. Introduction

The transendothelial recruitment of leukocytes from the circular bloodstream to the site of inflammation represents a key event during inflammatory processes. Among a wide variety of cell signaling molecules, chemokines are the main drivers in orchestrating the transendothelial movement of immune cells by mediating through their G-protein coupled receptors (GPCRs) located on the surface of the respective leukocytes. Chemokines are small-size proteins (8–12 kDa) that comprise two major and two minor families: CC, CXC, CX3C, and C chemokines. Although the sequence homology is only as low as 20%, the presence and formation of the conserved cysteines provide the specific tertiary structure of all chemokines. Today, approximately 50 different chemokines and 20 different GPCRs have been identified, showing striking levels of promiscuity. Most chemokines interact with multiple receptors and vice versa, and only a few have monogamous properties [1,2]. However, most chemokines favor one class of chemokine receptors, which is also the basis of their nomenclature system [3].

**Figure 1:**
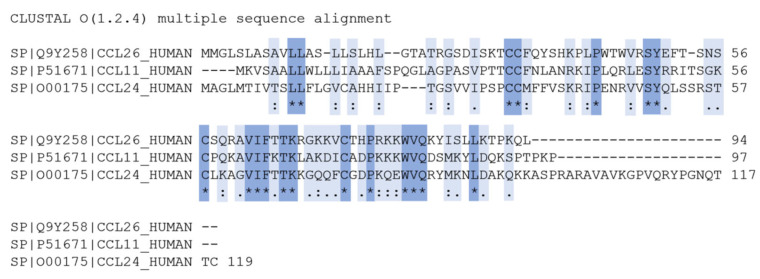
Sequences homology of eotaxins CCL11 and CCL24 to CCL26; “*” (asterisk): Positions with a single, fully conserved residue; “:” (colon): Conservation between groups of strongly similar properties (scoring > 0.5 in the Gonnet PAM 250 matrix); “.” (period): Conservation between groups of weakly similar properties (scoring ≤ 0.5 in the Gonnet PAM 250 matrix).

CCL26, also referred to as eotaxin-3, is a proinflammatory CC chemokine that belongs to the three-membered eotaxin family (together with CCL11/*eotaxin* and CCL24/*eotaxin-2*) [4]. Although the three proteins share sequence homologies of less than 40% (see Figure 1) and are also chromosomally distinct, they have been determined as the eotaxin family due to their unique ability to induce chemotaxis of eosinophils via CCR3 activation [5]. The CCR3 receptor is the most abundantly expressed receptor on eosinophils but can also be found on basophils and T-lymphocytes [6,7,8]. The expression of CCR3 is mainly enhanced upon IL-3 stimulation [9]. The exhibition of distinct temporal and spatial expression patterns of the three eotaxins under (patho-)physiological conditions suggests an independent role for each eotaxin in recruiting leukocytes. Significantly elevated gene levels of CCL26 are found in eosinophilia-associated diseases, such as eosinophilic esophagitis and eosinophilic gastritis, where transepithelial migration of eosinophils is one of the key steps for pathogenesis [10,11,12]. Infiltrated eosinophils release proinflammatory, tissue-damaging, cytotoxic compounds, such as major basic proteins 1 and 2, eosinophil-derived neurotoxin, peroxidase, and eosinophilic cationic proteins [13].

It has been reported that CCL26 must be membrane-bound to cell surfaces to mediate the transepithelial migration of eosinophils [14]. Although both secreted and membrane-bound CCL26 are present in the case of vascular endothelial cells, only the surface-associated form was critical for transendothelial migration [15]. The efficient retainment of CCL26 on cell surfaces is mainly provided by glycosaminoglycans (GAGs) and vastly contributes to eosinophilia. The importance of chemokine/GAG-binding for leukocyte trafficking has been shown for various chemokines in vivo and in vitro and has also become subject to diverse therapeutic tackling mechanisms.

**Figure 2:**
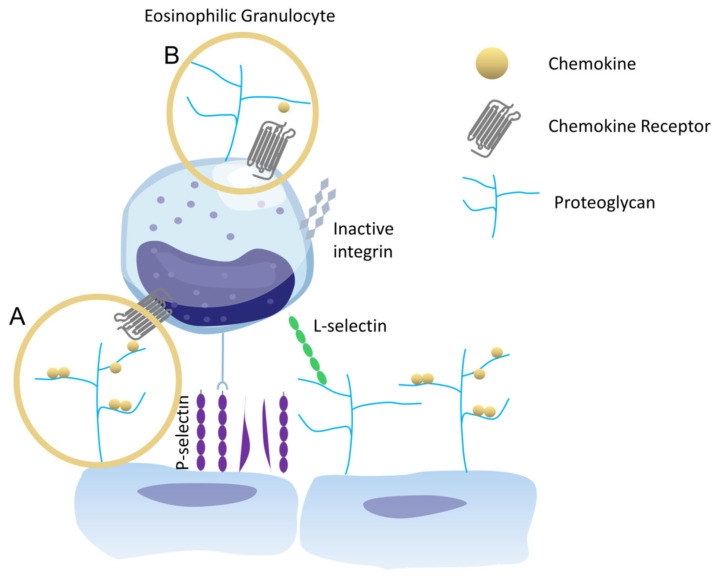
Proteoglycans (PGs) located on the endothelium (**A**), postulated as “*trans*” activity, and PGs located on the surface of eosinophils (**B**), postulated as “*cis*” activity of GAGs/PGs—with respect to with G-protein coupled chemokine receptors on the immune cell—orchestrate the migration of these cells to inflammatory sites.

When bound to a core protein, GAGs—as part of proteoglycans (PGs)—facilitate chemokine-mediated transmembrane leukocyte migration by interacting as so-called chemokine co-receptors (see Figure 2). These unbranched, linear, and (except for hyaluronan) highly sulfated polysaccharides are composed of repeating disaccharide units formed by uronic acid (L-iduronic acid or D-glucuronic acid) and an amino sugar (D-glucosamine or D-galactosamine) [16]. The linkage can be formed via α- or β-glycosidic bonds, distinguishing their relative stereochemistry based on the anomeric positioning and the stereogenic center furthest from the carbonyl group [17]. The family of sulfated GAGs comprises five members: Chondroitin sulfate, dermatan sulfate, keratan sulfate, and heparin heparan sulfate (HS), where the latter is the predominant one on mammalian cell surfaces. Hyaluronan is the only representative of non-sulfated GAGs. The variability of the sulphation patterns (C-4, C-6, and/or non-acetylated N of the amino sugar), together with the geometry of the linkage and the varying length (roughly 10–100 kDa), provides high degrees of structural possibilities and flexibility. GAGs can be found covalently bound to core proteins as PGs, forming a relatively thick layer—the glycocalyx—on the surface of vascular endothelial cells [18]. Not only does it function as a static permeability barrier, but the glycocalyx is also highly selective and undergoes drastic changes under pathological conditions [19].

At physiological pH, all carboxylic acid and sulfate groups are deprotonated, providing GAGs with a highly negative charge density [16]. The binding of chemokines to GAGs is thus driven mainly by electrostatic interactions, occurring between positively charged amino acids (arginine, lysine, and to a lesser extent histidine) and the highly sulfated S-domains of the polysaccharide. The specificity of protein-GAG interactions results from van der Waals and hydrogen bonding forces between the less charged regions of the polysaccharide and respective amino acids of the protein [20].

The binding of chemokines to GAGs facilitates the formation of an immobilized chemotactic gradient that provides the communication between the chemokine and the corresponding receptor, which features the essential leukocyte infiltration in inflammatory events into the affected tissues [21,22]. Studies showed, for several chemokines, that GAG-binding-deficient mutants could not mediate immune cell recruitment in vivo in contrast to their wildtype counterparts [23,24,25].

It, therefore, seems reasonable to develop glycan-targeted therapeutic approaches, such as GAG-mimetics and GAG-directed antibodies [26,27,28]. Another GAG-targeting approach that we have described previously involves engineered chemokines as so-called “dominant-negative” decoys with increased GAG-binding affinity and knocked-out GPCR-binding potency [29]. Regardless of the therapeutic pathway, investigating the mode of interaction is an essential prerequisite for designing interfering agents in the triangular GAG/chemokine/GPCR axis. For several chemokines, detailed information concerning the localization of the chemokine’s GAG-binding residues and their possible recognition pattern is available, while the data for CCL26 are very limited [30].

Syndecans (SDC) and glypicans (GPC) are both HS-based PGs that have pivotal roles in wound healing, inflammation, and stages of tumor progression. Previously published for neutrophils and monocytes [31], the existence and contribution of leukocyte-located heparan sulfate proteoglycans (HSPG) in CXCL8- and CCL2-mediated immune cell migration was confirmed for the first time.

The presence and role of eosinophilic PGs (Figure 2B) have not been investigated until today. Here, we report the engineering and in vitro characterization of a panel of CCL26 alanine-mutants studied according to their altered GAG-binding affinities and chemotactic activities relative to their wildtype counterpart. By this means, we were able to identify amino acid residues of CCL26 crucial for HS-binding. Further, we tested the mutants in cell-based migration assays to investigate the presence of basal proteoglycans PGs on the eosinophilic cell surface and their influence on chemokine-mediated cell migration. Our results revealed that K60A in the C-terminal α-helix of CCL26 is the most critical residue for HS-binding, whereas β-sheet located mutations did not alter the binding affinities significantly. We could further verify the presence of HS-based PGs on the eosinophilic cell surface, and that binding of CCL26 to these membrane-bound molecules is mandatory for and directly influences its chemoattracting ability for mediating eosinophil migration.

## 2. Results

### 2.1. CCL26 Structure Analysis and Engineering

**Figure 3:**
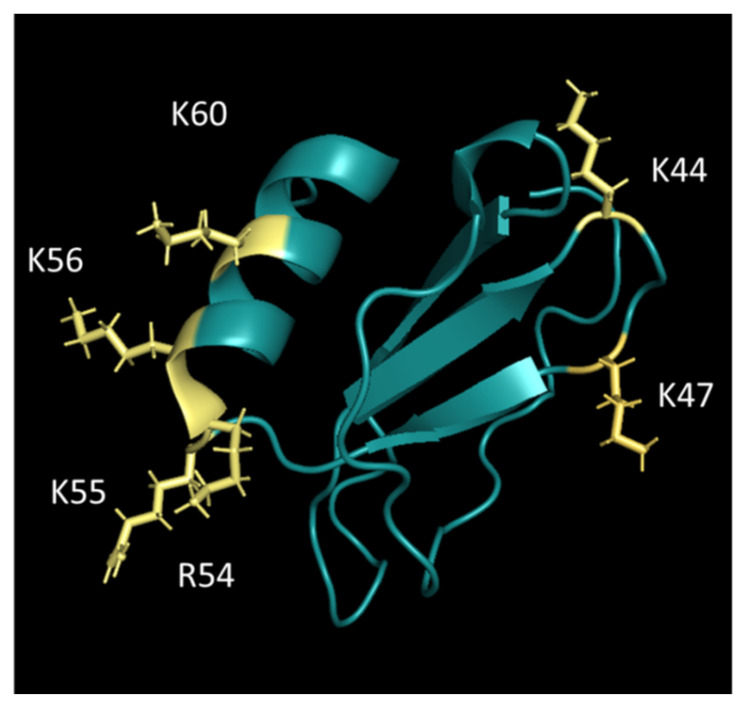
Design of GAG-binding knock-out alanine mutants of CCL26 (pyMOL.org); positively charged amino acids (K47A, K44A, R54A, K55A, K56A, and K60A) that have been replaced by alanine are highlighted in yellow.

Based on the results of already-identified GAG-binding sites of CC and CXC chemokines [30], we designed alanine mutants using the structure of monomeric human CCL26 (UniProtKB—Q9Y258). Solvent-exposed lysine and arginine residues were the prime candidates for site-directed alanine replacement mutagenesis (see Figure 3). This is due to the fact that GAG-binding is typically restricted to surface areas of proteins; only in some cases does a large conformational change occur following the interaction with GAGs.

### 2.2. Recombinant Protein Expression and Purification

In the course of this study, we expressed wildtype CCL26 and a panel of GAG-binding knock-out mutants in the *E. coli* BL21Star(DE3) host expression system (Table 1). The sequence of wtCCL26 and its mutants is depicted in Supplementary Data S1. The alanine mutations have been selectively inserted into specific sites in the sequence of CCL26 by a site-directed mutagenesis approach (see Section 3). In this way, we created four CCL26 variants with mutations in the C-terminal α-helix (K60A, K55A/K56A, R54A/K55A/K56A, and R54A, highlighted in Figure 3), as well as two proteins with a point mutation in the β-sheet (K44A and K47A). The CCL26 ΔP53-L71 truncation mutant provides unique features, as the complete α-helix is truncated in this mutant. A-helical mutations were of great interest based on previously published data of chemokines, where the GAG-binding site was already located in the C-terminal α-helix [30,32]. In terms of purity, the processed recombinant proteins were analyzed on 15% SDS PAGE gels and silver-stained according to the EMBL protocol (see Supplementary Data S2A,B). In addition, Western blot analysis was carried out to detect degradation products (see Supplementary Data S2C). The obtained proteins showed a purity of greater than 95% without degradation products. After purification of the mutants, structural characterization was performed by far-UV circular dichroism spectroscopy by which signals for properly folded α-helices and β-sheets were found except for the CCL26 ΔP53-L71 mutant, which has a reduced content in the α-helical structure, with a reduced turn, but also in lower β-sheet content (see Supplementary Data S3 and S4). All proteins were, in general, quite stable when unfolded using Guanidine-hydrochloride (GdmHCl). Unfolding transition (UT) points between 3.0 and 4.2 M GdmCl concentrations were found (see Supplementary Data S5 and S6).

### 2.3. Determination of Glycosaminoglycan-Binding Affinities by Isothermal Fluorescence Titration (IFT)

To characterize GAG-binding affinities of the proteins, unfractionated HS was used as the most relevant GAG ligand in vivo. Since it is also the most frequently occurring type of GAGs on cell surfaces and in the extracellular matrix (ECM) as part of a PG [16], it serves as a suitable prototype for chemokine/GAG-related experiments. However, it should be borne in mind that the GAGs used here in vitro are not identical to glycans in the human body.

The intrinsic fluorescence of tryptophan residues in three positions of the proteins (W21, W23, and W57) was used as a highly sensitive sensor for binding externally added GAGs in isothermal fluorescence titration (IFT). The stepwise addition of GAGs to a protein solution of a defined concentration resulted in a dose-dependent quenching of the probes’ fluorescence caused by the impaction of the tryptophan emission due to conformational rearrangements following ligand binding. The manner of the dose-dependent fluorescence intensity decrease (slope, stiffness, saturation behavior) reveals detailed information concerning the strength of the interaction. Not requiring genetic or chemical ligand/protein modifications and the fact that GAG ligand binding can be measured using very low chemokine quantities provide a considerable advantage of this technique.

**Figure 4:**
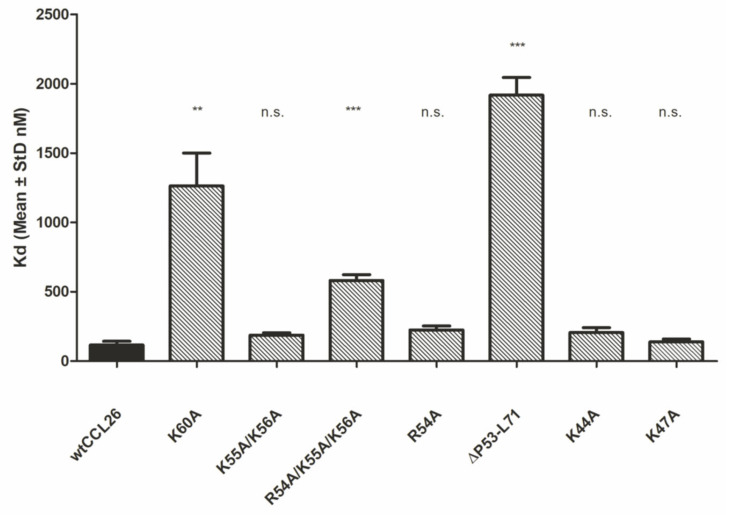
Kd values of CCL26 mutants (α-helix and β-sheet mutations) to heparan sulfate in isothermal fluorescence titration (IFT), ** *p* < 0.01, *** *p* < 0.001 was considered statistically significant (n.s.: statistically not significant).

**Figure 5:**
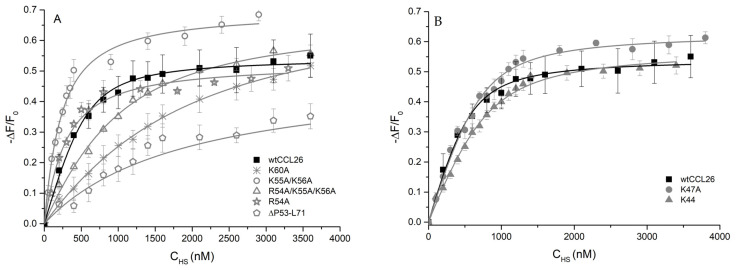
Heparan sulfate-binding isotherms of CCL26 mutants in α-helix (**A**) and β-sheet (**B**). Wildtype isotherms are shown in black. On the y-axis, the relative change in fluorescence intensity following ligand addition is displayed: ΔF = F (fluorescence emission at a certain ligand concentration)—F0 (fluorescence emission in the absence of ligand).

Figure 4 depicts the Kd values determined by IFT of HS to a 700 nM protein solution dissolved in phosphate-buffered saline. As described in Section 3, binding isotherms were analyzed as bimolecular association reactions by non-linear regression. Significant differences have been obtained from three out of seven potential CCL26 mutants (Figure 5). The point-mutated CCL26 K60A mutant induced an 11-fold decrease in the chemokine’s affinity for the HS ligand compared to wtCCL26 (1262.67 nM ± 237.62 to 116.28 nM ± 27.27). Only the truncated mutant CCL26 ΔP53-L71, which lacks the entire α-helix, showed an even lower binding affinity to HS (17-fold decreased affinity; 1917.59 nM ± 128.03). The Kd value of the α-helical triple-mutant CCL26 R54A/K55A/K56A was determined to be 5-fold decreased compared to the wildtype protein (581.29 nM ± 41.62). This could be possibly attributed to the change in pI value, as a reduced overall charge density accompanies the mutations (9.98 to 10.22; see Table 1). β-sheet-located mutations (in CCL26 K44A and CCL26 K47A) did not lead to a significant change in the binding of the mutants to HS, compared to wt CCL26 (206.06 nM ± 35.68 and 139.32 nM ± 19.45 to 116.28 nM ± 27.27; see Figure 1, right). CCL26 R54A depicted as little of a change in the GAG interaction as the other two mutations (225.35 nM ± 28.7). These results suggest the crucial role of α-helical structures of CCL26 in GAG-binding, but not a strong involvement of the β-sheet residues. Based on the outstanding influence of the alanine replacement mutation in position 60 on HS-binding, critical GAG-binding features can be attributed to this amino acid and its structural environment.

### 2.4. Quantitative Real-Time PCR of Proteoglycans on RNA Level

The cell surface PG expression (i.e., syndecans and glypicans) has been investigated for lymphoid cells, macrophages, and monocyte-derived dendritic cells and are known to be altered in different pathophysiological conditions [33,34]. Knowing the PG expression pattern on immune cells would therefore be a valuable prerequisite for developing novel GAG-targeting therapeutic approaches. There is, however, currently very limited knowledge about the PG expression on immune cell surfaces and thus about their involvement in immune and inflammatory processes.

**Figure 6:**
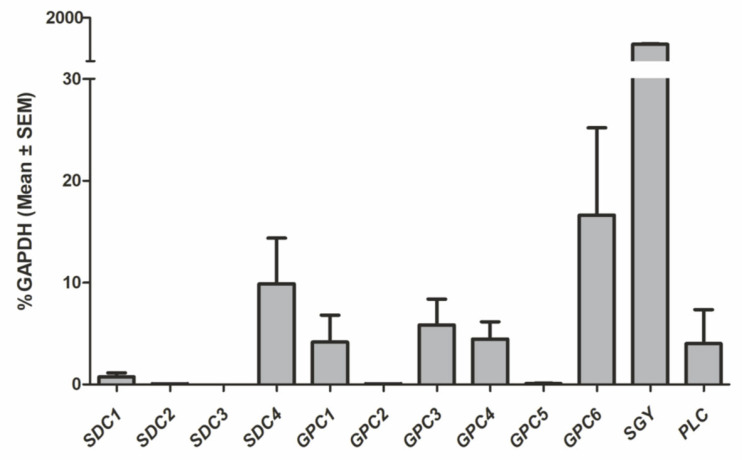
Gene expression profile of syndecans (*SDC1-4*), glypicans (*GPC1-6*), and perlecan (*PLC*) in eosinophils in comparison to housekeeping gene *GAPDH* RNA expression (set as 100%); elevated levels were determined for *SDC4*, *GPC1*, *3*, *4*, *6*, *serglycin*, and *perlecan*; mean CT used in the analysis of relative gene expression levels.

We have characterized the PG expression profile of blood-derived eosinophils by qPCR analysis. The analysis included the gene expression on RNA level of twelve different PGs of freshly prepared eosinophils (data are displayed relative to *GAPDH* expression). In Figure 6, the expression levels of HS-proteoglycans are shown. Besides syndecans (*SDC*) *1-4* and glypicans (*GPC*) *1-6*, we also examined gene expression levels of serglycin and perlecan PGs. Threshold cycles (C_T_) of the individual genes were defined as the cycle number at which fluorescence exceeds an empirically determined value and are illustrated in Figure 6.

Among all tested syndecans, the highest gene expression levels were found for *SDC4* (9.88% ± 4.48), while the other three members of the family showed little (for *SDC1 0.73% ± 0.41*) or no gene expression (*SDC2* and *SDC3*) in eosinophils. HS-based PG *SDC4* RNA expression on eosinophils has already been reported in the past [35] relating to elevated levels in eosinophil-related airway inflammations. It has been reported that the blocking of *SDC4* signaling in asthmatic mice results in a diminished asthmatic phenotype compared with control animals, suggesting an essential role of immune-cell-located glycans as modulators in chronic inflammations [36]. Perlecan and glypicans 1, 3, and 4 are all in the same range with threshold C_T_ values of approximately 5% of *GAPDH* expression, while *GPC2* and *GPC5* have not been detected in qPCR analysis. 

Interestingly, serglycin was found to be the PG with the highest expression level (1698% ± 3.49), exceeding *GAPDH* RNA expression 1000-fold. It has been described as the most dominant PG species in most immune cells and is a pivotal factor in immunological processes. Since the majority of serglycin expression levels were studied in mast cells, the investigation of its particular role in (eosinophilic) granulocytes biology is missing so far. Chondroitin sulfate-4 has been reported as the major GAG component in eosinophil-derived serglycin, where it is found in secretory granules [37,38].

### 2.5. Eosinophil-Based Migration Assays

To compare the chemotactic ability of wildtype CCL26 and its mutants, an in vitro Boyden chamber chemotaxis assay was performed using freshly prepared human eosinophilic granulocytes. Chemotaxis was evaluated by the CI (chemotactic index), which is defined as the ratio between the total number of migrated eosinophils and the number of nonspecifically migrated cells towards the buffer.

**Figure 7:**
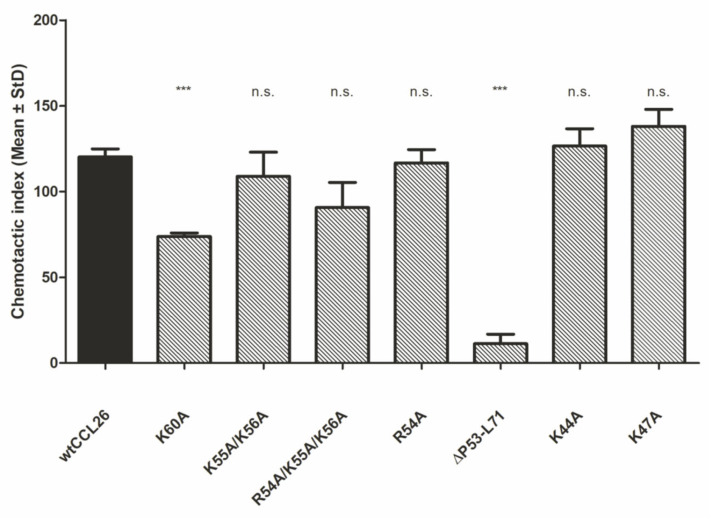
Results from Boyden chamber migratory assay of eosinophils towards 150 nM concentration of GAG-binding knock-out mutants of CCL26; *** *p* < 0.001 was considered statistically significant; n.s., non-significant.

Chemotactic activity was found for wtCCL26 and all mutants (CI ranging between 109 and 138), except for CCL26 ΔP53-L71 (CI 11.22 ± 5.58), which was completely deficient in the recruitment of eosinophils. All the other chemokines were able to induce high levels of eosinophil migration at 150 nM (data from 15 mM and 1500 nM not shown). We further observed a significant trend, where decreasing GAG-binding affinities of the chemokine mutants are accompanied by reduced migration behavior of eosinophils toward the proteins (CCL26 K60A and CCL26 ΔP53-L71, Figure 7). This indicates that the leukocyte migration is GAG-dependent in this in vitro setup, suggesting the presence of GAGs on eosinophils. We have recently described this phenomenon, i.e., the involvement of GAGs on immune cells in chemotaxis, for neutrophil and monocyte migration in response to CXCL8 and CCL2, respectively [31]. The newly discovered chemotactic involvement of PGs on the surface of immune cells was termed the “*cis* activity” of GAGs (i.e., being located on the same cell as the chemokine receptor), compared to the accepted “*trans* activity” according to which the chemokine receptor and its GAG co-receptor are located on different cells (see Figure 2). The *cis* activity means that leukocyte PGs function as an additional stimulator (co-receptor) for immune cell migration in the interplay with the likewise leukocyte-located GPCRs [39].

**Figure 8:**
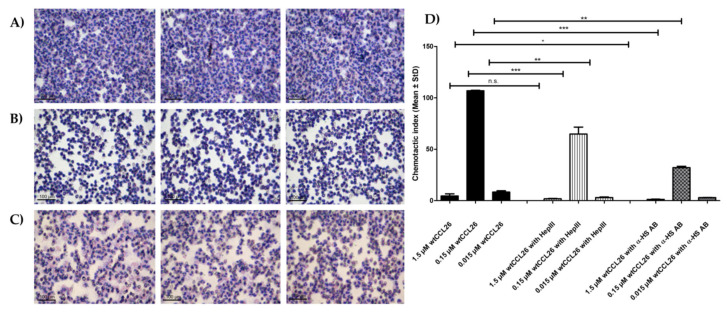
**Left**, migrated eosinophils for (**A**) untreated, (**B**) heparinase-III-treated, (**C**) anti-HS-antibody masked eosinophils in response to 150 nM CCL26 visualized via 40× magnification. **Right**, (**D**) chemotactic activity of untreated, heparinase C (hepIII), and with anti-heparan sulfate antibody (α-HS AB)-treated eosinophils in response to wildtype CCL26 (1.5 µM–0.15 µM–0.015 µM); * *p* < 0.05, ** *p* < 0.01, *** *p* < 0.001 was considered statistically significant (n.s.: statistically not significant).

To further investigate this hypothesis, we enzymatically digested isolated eosinophils with heparinase III (hepIII) prior to their use in the chemotaxis assay in order to rid the cells of their glycan coating (“*shedding*”). As can be seen in Figure 8, after removing the digested GAG fragments by centrifugation, the GAG-shed eosinophils were much less responsive to the chemotactic activity of wtCCL26 (1500 nM CI 1.72 ± 0.42; 150 nM CI 64.75 ± 6.77; 15 nM CI 30.3 ± 0.51). The untreated control chemotaxis depicted, in contrast, CI values of 4.57 ± 2.05 for 1500 nM, 106.8 ± 0.53 for 150 nM, and 8.28 ± 1.31 for 15 nM. As additional proof of this hypothesis, we incubated eosinophilic granulocytes with an anti-HS antibody and again measured the chemotactic activity of wtCCL26. The anti-HS antibody also led to significantly decreased eosinophilic chemotaxis induced by wtCCL26 (1500 nM 1.24 ± 0.22; 150 nM CI 32.13 ± 1.31; 15 nM CI 2.91 ± 0.25). Taken together, these results strongly indicate the presence and involvement of PGs on the surface of eosinophils.

### 2.6. Chemotaxis of Eosinophils Incubated with an Anti-Serglycin Antibody

Based on the results from the qPCR analysis, chemotaxis experiments, including an anti-serglycin antibody, were performed to investigate the impact of serglycin in CCL26-induced eosinophilic cell migration. Eosinophils were again freshly isolated from female Caucasian whole blood and subsequently used for Boyden chamber assays. For this purpose, the lower compartment of the Boyden chamber contained 150 nM wtCCL26, as it was the most active chemokine concentration identified in previous experiments (Figure 8). The cells were preincubated with a monoclonal serglycin antibody for 20 min at three different concentrations (0.1, 1.0, and 10 µg/mL) and then applied to the upper chamber in triplicate. After incubation of 120 min, migrated eosinophils were visualized on a 5 µm porous membrane as described previously.

**Figure 9:**
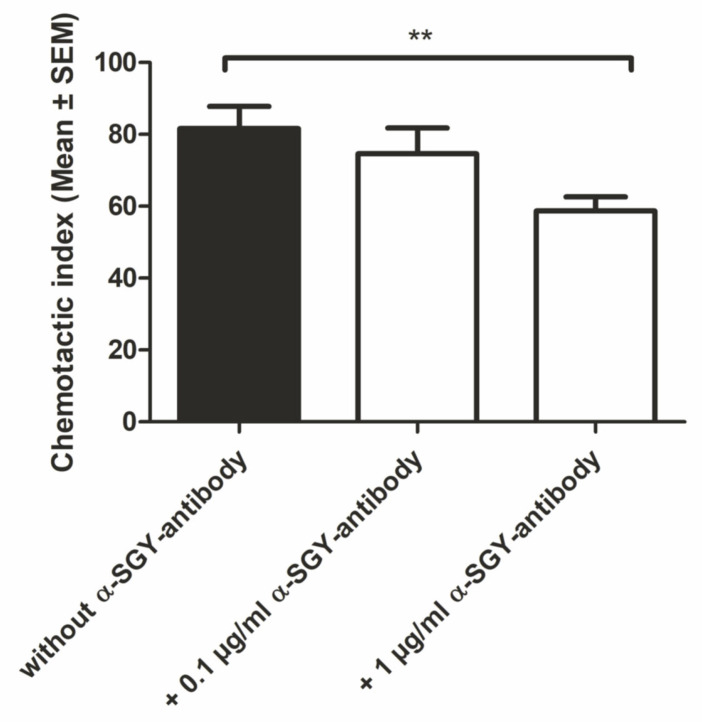
Chemotactic response of 150 nM CCL26 towards eosinophils preincubated with anti-serglycin antibody in three different concentrations compared to non-treated cells; ** *p* < 0.01 was considered statistically significant; n.s., non-significant.

While no difference was observed in the migration behavior at an antibody concentration of 0.1 µg/mL, significantly decreased migration activity of CCL26 towards eosinophils treated with 1 µg/mL serglycin antibody was observed compared to non-treated cells (Figure 9). These results suggest that serglycin, the PG with the highest expression level in eosinophils, directly impacts the CCL26-induced eosinophilic cell migration.

## 3. Materials and Methods

General laboratory and reagents were purchased from Sigma-Aldrich (Poole, UK) unless stated otherwise. Primers used for mutagenesis were ordered from ThermoFisher (Waltham, MA, USA). Unfractionated HS was purchased from Iduron Laboratories (Cincinnati, OH, USA).

### 3.1. Mutagenesis

The synthetic *E. coli* codon-optimized huCCL26 gene in pJ411 expression vector was purchased bfromy ATUM (Newark, CA, USA). Using huCCL26 as a template, seven CCL26 “knock-out” mutants (decreased binding affinity to GAGs) were prepared by site-directed mutagenesis with the help of complementary oligonucleotide pairs encoding the intended mutations (see Table 2), a Q5 high-fidelity polymerase (NEB, Ipswich, MA, USA), and dNTPs (NEB). After using Eppendorf’s mastercycler gradient for PCR, the original plasmid was digested with Dpn1 (NEB), and the remaining modified ones were treated with kinase (NEB) and ligase (NEB) enzymes as per the manufacturer’s instructions. The plasmids were then transformed into *E. coli* Top10 for plasmid amplification and subsequently isolated using Qiagen Midiprep kits according to the manufacturer’s directions. For verifying the presence of the intended mutations, the obtained plasmids were sent to LGC Genomics (Berlin, Germany) prior to expression and purification. The plasmid for ΔP53-L71 was also directly designed in the pJ411 expression vector and ordered via ATUM.

### 3.2. Fermentation and Purification of CCL26 and Mutants

Verified DNAs were transformed in calcium-competent *E. coli* BL21star(DE3) cells, and the resulting colonies were used for an overnight culture in Luria Bertani medium (20 g/L; 50 µg/mL kanamycin sulfate). The cultures were incubated for 16 h at 37 °C and 225 rpm. They were then diluted 1:1000 in LB medium and incubated again under the conditions as per the description above. After reaching an optical density (600 nm) > 0.65, the protein expression was induced by adding IPTG (isopropyl-b-D-thiogalactopyranoside) to a final concentration of 0.5 mM. After another 3 h, the cells were harvested at 6000 rpm for 10 min, and the pellet was stored at −20 °C. All proteins were then purified, as reported in Falsone et al., 2013, but under non-endotoxin-free conditions [29]. In brief, the proteins were all purified in a three-dimensional column chromatographic process, starting with liquid chromatographic purification (column material Fractogel EMD SO_3−_, GE Healthcare, Uppsala, Sweden), followed by a denaturing rpHPLC step (Hi-bar 250-25 column with LiChrosphere 100 RP-18 12 µM, Merck, Darmstadt, Germany). The proper refold was ensured by a subsequent SP Sepharose column (GE Healthcare, Uppsala, Sweden) followed by dialysis against 1xPBS. The protein solutions obtained were concentrated to concentrations between 0.5 and 2.5 mg/mL using a 3 kDa cellulose membrane centrifugation filter (Amicon Ultra-15, Merck-Millipore, Burlington, MA, USA). The purity of all proteins was examined with silver staining according to the European Molecular Biology laboratory (EMBL) protocol, and their identification was verified with Western blot analysis.

### 3.3. Structural Characterization: Far-UV Circular Dichroism Spectroscopy

As a valuable method for analyzing 3-D structures of the recombinant proteins, circular dichroism spectroscopy was used. This technique exploits the unequal absorption behavior of left-handed and right-handed circularly polarized light when interacting with asymmetric molecules. Different secondary structural elements provide characteristic CD spectra. While α-helical formations typically show negative bands at 222 nm and 208 nm and a positive band at 193 nm, antiparallel β-sheets are characterized by a negative band at 218 nm and a positive one at 195 nm. Unstructured proteins with disordered structures have been shown previously [40].

Proteins were analyzed at 700 nM in the respective Quartz cuvettes (Hellma, Baden Germany) in 1xPBS. Before starting with the first protein-containing sample, the system was blanked against 1xPBS. The spectrum was recorded in triplicates on a Jasco J-1500 instrument (start/end: 250 nm/190 nm; sensitivity: 500 mdeg; scan speed 50 nm/min; data pitch 0.2 nm). Analysis of the received data was performed using the BeSTsel online tool (BeStSel, Budapest, Hungary ). By inserting the raw data (MRE units, concentration of the protein sample, number of residues and pathlength), the software allows an evaluation of the particular content of α-helices, β-sheets (antiparallel, parallel), turns, and unstructured residues.

### 3.4. Isothermal Fluorescence Titration (IFT)

In order to measure binding affinities in protein-GAG complexes without modifying either of the two parameters on a chemical or genetical level, IFT experiments were performed. The binding of stepwise titrated GAGs results in a dose-dependent decrease (“quenching”) of the fluorescence intensity as a consequence of the HS-induced structural rearrangement of the protein. Titrations were carried out on a Fluoromax Spectrofluorometer (Horiba, Kyoto) according to Gerlza et al. (2014) [41]. The spectra were recorded at a temperature of 20 °C by using an external water bath and at a speed of 500 nm/min. HS was purchased from Celsus Laboratories Inc. (Cincinnati, OH, USA), and the respective 100 and 500 µM stock solutions were prepared in 1x phosphate-buffered saline. 0.7 µM of CCL26 and mutant solutions were equilibrated for 30 min prior to the fluorometric scans. Furthermore 100 and 500 nM HS ligand aliquots were stepwise added to the solutions, and after 1 min of incubation, the spectra were recorded. Three independent measurements were performed for each mutant, and the normalized mean changes in the fluorescence intensity (−ΔF/F0) were plotted against the corresponding HS concentration. The data were evaluated as published previously [41] to be able to define the Kd value (dissociation constant) by analyzing the binding isotherms by non-linear regression (Origin Microcal Inc., Northhampton, MA, USA) for all proteins.

### 3.5. Preparation of Human Eosinophils

Human whole blood was collected from healthy donors (female, Caucasian) through venepuncture using K3EDTA vacuettes (Greiner bio-one, Kremsmünster, Austria). Eosinophils were directly isolated with the EasySep^TM^ direct human eosinophil isolation Kit (StemCell, Cologne, Germany) as per the manufacturer’s description, and diluted to a final concentration of 1 × 10^6^ cells per mL in 1xPBS 1 mM EDTA.

### 3.6. RNA Isolation and qPCR of Proteoglycans

To isolate the containing RNA, the eosinophil pellet was resuspended in 1 mL Trizol-RNA extracting reagent and incubated for 10 min at room temperature. After adding 200 µL of chloroform, the mixture was vortexed for 20 s and centrifuged for 15 min at 12,000× *g*. The upper, aqueous RNA-containing phase was carefully pipetted into a fresh tube, where 500 µL of isopropanol was added. After an incubation time of 10 min at room temperature, the solution was centrifuged (10 min, 12,000× *g*). The remaining RNA pellet was washed with 75% EtOH and once more centrifuged at 7500× *g* for 5 min. The pellet was dried for approximately an hour and eventually resuspended in 30 µL of nuclease-free water. The purity and quantity of the isolated RNA were examined via 260/280 nm on a NanoDrop Spectrophotometer.

For transcription in cDNA, the “High Capacity cDNA Reverse Transcription Kit” (Applied Biosystems) was used. Primers for mRNA expression analysis of the respective PGs are listed in Table 3. Melting points of the primer are in the range of 5 °C for each primer pair. The SYBR^®®^ green PCR master mix was used for qPCR analysis on the StepOnePlus Real-Time PCR System (Applied Biosystems). Furthermore, 20 µL of cDNA and 450 µL of nuclease-free water were added to 520 µL of the master mix and pipetted into a 96-well plate. Corresponding primer pairs were added to a final concentration of 0.25 µM to each well. As a control, primers for housekeeping glyceraldehyde 3-phosphate dehydrogenase (GAPDH) genes were also applied to the plate. The experiments were carried out in triplicate (1: 95 °C, 10 min, 2: 95 °C, 15 s, 3: 60 °C, 60 s; 40 cycles of 2. + 3.).

Data evaluation is based on the relative gene expression of particular PGs to the housekeeping GAPDH gene level using threshold cycles (CT)-determined endpoints (see Equations (1) and (2)). CT is inversely related to the initial amount of the target, meaning a low CT value indicates a high number of amplicons. A ΔCT comparative quantification algorithm was used to determine the expression level of our genes of interest. The following equations were used for evaluating the gene expression amount, where GAPDH was set to 100%:

ΔCT = [mean CT of sample] − [mean CT of GAPDH],(1)

(2) $$\%\;\mathrm{GAPDH}=\frac{100}{2^{-\Delta \Delta \mathrm{CT}}}$$

### 3.7. Eosinophil Chemotaxis

Chemotaxis assays were used to investigate the chemotactic response of eosinophils to CCL26 and mutants using a 48-well Boyden Chamber (Neuroprobe, Gaithersburg, MD, USA) and PVP-free polycarbonate membranes (pore size: 5 µM, Neuroprobe). Eosinophils were placed in the upper compartment (50 µL) of the chamber and were allowed to migrate through the microporous membrane towards proteins in the lower compartment (29 µL), where triplicates of CCL26 and mutants were applied in different concentrations (0.015/0.15/1.5 µM). The chamber was incubated for 90 min at 37 °C in a 5% (*v*/*v*) CO_2_ humified incubator. After removing the filter from the chamber and washing away non-migrated cells from the filter with 1xPBS, the migrated cells were fixed with MeOH and stained with Hemacolor solutions (Merck). The chemotactic index was examined by counting the migrated cells at 40× magnification in five randomly picked fields per well. Considering the background migration, the chemotactic index was calculated for CCL26 and mutants.

#### Treatment of Cells

Heparinase III (hepIII) was expressed and purified as stated in [31] Section 2.4. After isolating freshly prepared eosinophilic granulocytes from the whole blood of healthy donors, the cells were divided into three fractions. The first was incubated with hepIII (45 µg/mL) for 37 °C (water bath) and subsequently centrifuged (5 min, 500× *g*) to remove digested GAG fragments. Fractions 2 and 3 were also incubated and centrifuged under the same conditions but without externally added hepIII. All cell pellets were resuspended in the respective amount of buffer. Moreover, 10 µg/mL of the HS-masking antibody (Amsbio) were directly added to eosinophilic fraction 2 prior to the assay. Furthermore, 95% viability of the cells was determined for all conditions by analyzing them under the microscope.

### 3.8. Statistics

The statistical analysis was determined by Student’s *t*-test using GraphPad Prism 8 software. For chemotaxis experiments, all conditions were repeated thrice in three independent wells with 5 photos of each well. Differences resulting in *p*-values < 0.05 (*), <0.01 (**) and <0.001 (***) were considered to be statistically significant.

## 4. Conclusions

The involvement of GAGs as endothelial-bound PG together with GPC-receptors on immune cells has been widely studied in chemokine-mediated cell migration of leukocytes. Neutrophils have been the focus of leukocyte-related inflammatory scenarios for several decades, whereas the subgroup of eosinophils has received less interest [42,43]. Furthermore, the interplay of neutrophil-attracting chemokines- such as IL-8, with their cognate chemokine receptors and co-interacting GAGs, has been widely studied in the last years [44,45,46,47]. GAGs participate in leukocyte recruitment by selectively binding and presenting chemokines to their corresponding immune-cell GPC receptors. However, much less is known about the presence and contribution of immune cell-located PGs in leukocyte migration, which was partly unraveled in this study.

Since the recognition of eosinophilia-associated chronic inflammation has been constantly increasing in recent years, eosinophil-related research has gained more and more attention [48,49]. In eosinophilic esophagitis (EoE), the presence of eosinophilia is a key feature for the diagnosis and pathogenesis of the disease. Patients suffering from eosinophilic esophagitis experience tissue damage caused by the high eosinophilic infiltration and reflect symptoms such as dysphagia, food impaction, and—especially for children—trouble swallowing [50]. Nevertheless, diagnostic guidelines and mechanical understanding for nonesophageal hypereosinophilic diseases such as eosinophilic gastritis, gastroenteritis, and colitis are still missing [51]. IL-5, IL-4, and IL-13 have been identified as the most upregulated genes by transcriptomic analyses in EoE, where the latter two are responsible for the induction of CCL26 expression [52,53]. CCL26 is the primary driver of eosinophilic infiltration in chronic diseases and could thus be a potential agent to interfere within these routes therapeutically.

In this study, we investigated the GAG-binding site of CCL26 by generating a panel of alanine-mutants via site-directed mutagenesis. Mutant K60A (α-helical point mutation) showed significantly decreased binding affinity to HS in IFT, whereas β-sheet located mutations did not show any differences compared to CCL26 wildtype. Moreover, for C-terminal α-helix truncated mutant CCL26 ΔP53-L71, only very low to no binding affinity was found in IFT. Considering the data from the other α-helical alanine-mutants, the results strongly indicate the involvement of α-helical amino acid K60 in GAG-binding, but not the contribution of β-sheet located residues. Since decreased binding affinities of CCL26 variants to HS were accompanied by significantly lowered chemotactic abilities of these mutants to mediate eosinophil migration, we further analyzed the presence and involvement of eosinophilic PGs in this axis.

We have previously published that PGs on neutrophils and monocytes contribute to CXCL8- and CCL2-mediated chemotaxis, respectively [31]. Here, we confirm that not only endothelial HS-based PGs are required for CCL26-induced chemotaxis of eosinophils, but that also eosinophil-located GAGs participate in this cell-signaling pathway. The binding of chemokines to HS facilitates the interaction with the cognate CCR3 on eosinophils and thereby enhances the eosinophil response to CCL26. The constantly increasing knowledge about the mode of action of CCL26/GPCRs and GAGs-related routes will provide powerful strategies for interfering in this multidimensional pathway.

## Figures and Tables

**Table 1:** Physical and chemical characteristics of wtCCL26 and its mutants that were expressed and purified; abbreviation: pI, isoelectric point.

Protein	Mutation Site	Molecular Weight *	pI *	Extinction Coefficient *^,^**	Purity Level ***
wtCCL26	-	8396.82	10.22	21.22	>95%
K60A	α-helix	8338.73	10.17	21.22	>95%
K55A/K56A	α-helix	8281.63	10.11	21.22	>95%
R54A/K55A/K56A	α-helix	8196.52	9.98	21.22	>95%
R54A	α-helix	8310.71	10.10	21.22	>95%
ΔP53-L71 ****	α-helix	6058.93	9.76	14.23	>95%
K44A	β-sheet	8338.73	10.17	21.22	>95%
K47A	β-sheet	8338.73	10.17	21,220	>95%

* Isoelectric point (pI) values are calculated using the ExPASy ProtParam online tool; ** Assuming all pairs of Cys residues form cystines; *** Estimated via silver-stained protein gels; **** amino acids 53 to 71 deleted, which correspond to the α-helix.

**Table 2:** List of primer sequences (fw, forward; rev, reversed) used for generating CCL26 mutants by site-directed mutagenesis.

Target		Primer Sequence
CCL26 K60A	fw	ATGGGTTCAGGCGTACATCAGCCTGTTGAAAAC
	rev	GTGGGTAGGCGCGTTCTT
K55A/K56A	fw	CCATCCGCGCGCGGCATGGGTTCAGAAG
	rev	CACCGTTTTTTCACACGTG
R54A/K55A/K56A	fw	CACCCATCCGGCCGCGGCATGGG
	rev	GTTCGCACCGTTTTTTCACAC
R54A	fw	CACCCATCCGGCGAAGAAATGGGTTCAG
	rev	CACACTTTTTTGCCACGC
K44A	fw	CTTCACGACCGCGCGTGGCAAAAAAAGTG
	rev	AATAACCGCACGCTGGCTG
K47A	fw	GCGTGGCAAAGCAGTGTGCACC
	rev	TTGGTCGTGAAGATAACC
Sequencing Primer	-	ATTCACGAGCAACAGCTGC

**Table 3:** List of primer sequences and gene accession numbers used for primer design for qPCR analysis of PGs; Abbreviations: SDC, syndecan; GPC, glypican; PLC, perlecan; GAPDH, Glycerinaldehyd-3-phosphate-dehydrogenase.

Target		Direction	Primer Sequence	Accession No.
Syndecan-1	*SDC1*	fw	GGAGCAGGACTTCACCTTTG	NM_002997.4
		rev	TACAGCATGAAACCCACCAG	
Syndecan-2	*SDC2*	fw	GCTGCTCCAAAAGTGGAAAC	BC049836.1
		rev	CAGCAATGACAGCTGCTAGG	
Syndecan-3	*SDC3*	fw	GAGCCTGACATCCCTGAGAG	NM_014654.4
		rev	CCCACAGCTACCACCTCATT	
Syndecan-4	*SDC4*	fw	GAGCCCTACCAGAGCATGAG	BC030805.1
		rev	CAGTGCTGGACATTGACACC	
Glypican-1	*GPC1*	fw	AGCGAGATGGAGGAGAACCT	BC051279.1
		rev	CTGAGTACAGGTCCCGGAAG	
Glypican-2	*GPC2*	fw	ACTGGGACACGACCTGGAC	NM_152742.3
		rev	CCCCAGAACCATCCCTTCTA	
Glypican-3	*GPC3*	fw	GGCAAGTTATGTGCCCATTC	KX533474.1
		rev	ATGTAGCCAGGCAAAGCACT	
Glypican-4	*GPC4*	fw	ATGGTGGCAGAGAGGCTAGA	AF030186.1
		rev	GGAACGAGAAATTCGTCCAG	
Glypican-5	*GPC5*	fw	AAGCCCAGTCTGGAAATCCT	AF001462.1
		rev	TCACAGTCCCCACTGCATTG	
Glypican-6	*GPC6*	fw	CACGTTTCAGGCCCTACAAT	AF105267.1
		rev	GTTCCAGCATTCCTCCTCGT	
Serglycin	*SGY*	fw	CGTCTGAGGACTGACCTTTTTCC	BC015516.1
		rev	CGTTAGGAAAGCCACTCCCAGAT	
Perlecan	*PLC*	fw	* VHPS-4370	
		rev		
Glycerinaldehyd-3-phosphate-dehydrogenase	*GAPDH*	fw	ATGTTCGTCATGGGTGTGAA	NM_001289746.2
		rev	GTCTTCTGGGTGGCAGTGAT	

* Purchased from Biomol GmbH; sequences are not revealed.

## Data Availability

Not applicable.

## Supplementary Materials

The following supporting information can be downloaded at: https://www.mdpi.com/article/10.3390/ijms23126519/s1.
