# Supplementary material for: The Role of Heparan Sulfate in CCL26-Induced Eosinophil Chemotaxis
Source: Int J Mol Sci. 2022 Jun 10;23(12):6519. doi: 10.3390/ijms23126519 (PMC9224159; doi:10.3390/ijms23126519)
Supplement: Supplementary file 1 [file ijms-23-06519-s001.zip › ijms-1754672-supplementary.pdf]

<u>**SUPPLEMENTAL MATERIAL**</u>

Supplementary Data S1. Sequences of wtCCL26 and its mutants

wtCCL26

TRGSDISKTC CFQYSHKPLP WTWVRSYEFT SNSCSQRAVI FTTKRGKKVC THPRKKWVQK YISLLKTPKQ L

CCL26 K60A (α-helical GAG knock-out mutant)

TRGSDISKTC CFQYSHKPLP WTWVRSYEFT SNSCSQRAVI FTTKRGKKVC THPRKKWVQA YISLLKTPKQ L

CCL26 K55AK56A (α-helical GAG knock-out mutant)

TRGSDISKTC CFQYSHKPLP WTWVRSYEFT SNSCSQRAVI FTTKRGKKVC THPRAAWVQK YISLLKTPKQ L

CCL26 R54A/K55A/K56A (α-helical GAG knock-out mutant)

TRGSDISKTC CFQYSHKPLP WTWVRSYEFT SNSCSQRAVI FTTKRGKKVC THPAAAWVQK YISLLKTPKQ L

CCL26 R54A K56A (α-helical GAG knock-out mutant)

TRGSDISKTC CFQYSHKPLP WTWVRSYEFT SNSCSQRAVI FTTKRGKKVC THPAKKWVQK YISLLKTPKQ L

CCL26 ΔP53-L71 (α-helix truncation mutant)

TRGSDISKTC CFQYSHKPLP WTWVRSYEFT SNSCSQRAVI FTTKRGKKVC TH

CCL26 K44A (β-sheet GAG knock-out mutant)

TRGSDISKTC CFQYSHKPLP WTWVRSYEFT SNSCSQRAVI FTTARGKKVC THPRKKWVQK YISLLKTPKQ L

CCL26 K47A (β-sheet GAG knock-out mutant)

TRGSDISKTC CFQYSHKPLP WTWVRSYEFT SNSCSQRAVI FTTKRGAKVC THPRKKWVQK YISLLKTPKQ L

Supplementary Data S2. Silverstain of wtCCL26 at three different concentrations. (A) depicts short silver staining, (B) overstaining and (C) detection with Western Blot. The product is >95% pure, only few additional bands are visible. Bands around 15 kDA correspond to dimer formation. Western Blot analysis confirmed that CCL26 is present as monomer and to a lesser extend as dimer

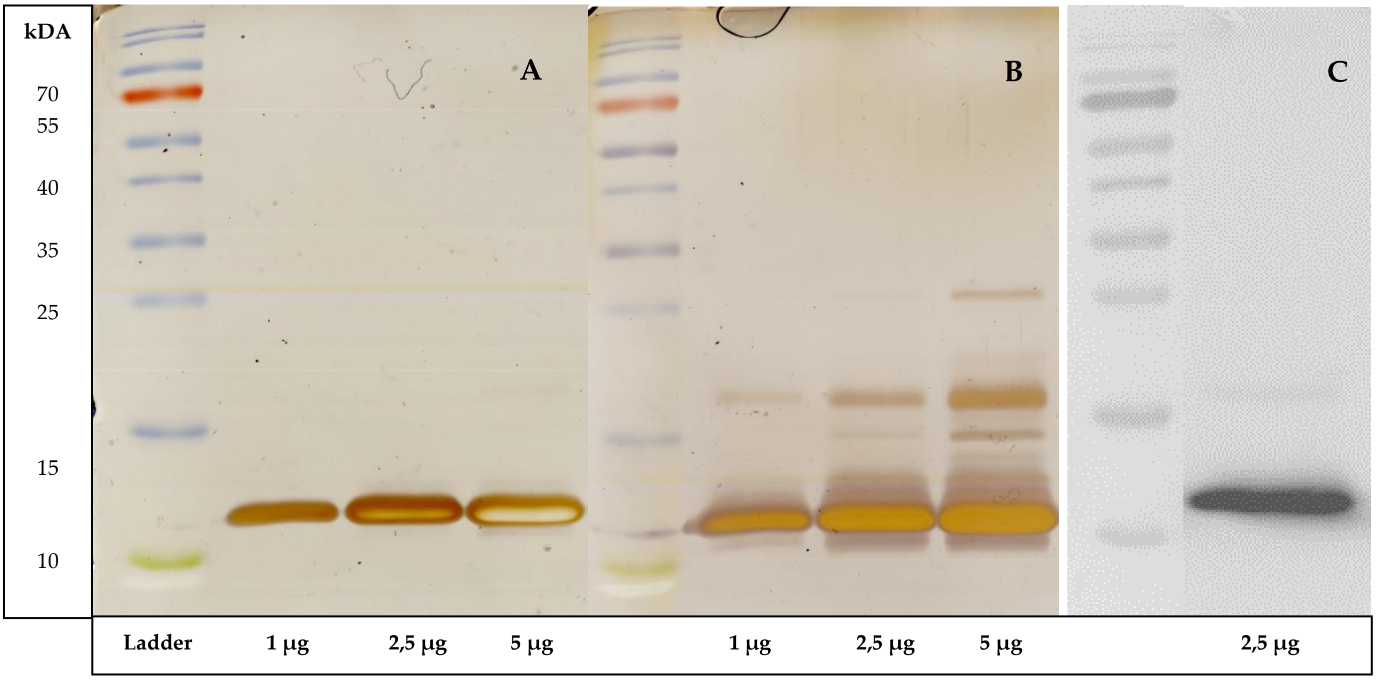

Supplementary Data S3. Spectra analysis results of circular dichroism (CD) spectroscopy data using BeSTSel software. CCL26 mutants depict a comparable secondary structure content compared to wtCCL26. The only outlier is the truncation mutant that is lacking the amino acids responsible for α-helix formation, which is in accordance with the obtained results

**Protein**	**Secondary structure content (in %)**				
	**Helix**	**Antiparallel**	**Parallel**	**Turns**	**Others**
wtCCL26	3,8	30,7	0,0	17,3	47,3
K60A	2,2	33,0	0,0	17,7	47,1
K55A/K56A	2,6	32,2	0,0	18,3	49,0
R54A/K55A/K56A	3,9	31,2	0,0	18,1	46,8
R54A	4,8	29,5	0,0	18,2	47,4
ΔP53-L71	1,6	27,7	0,0	12,1	58,4
K44A	4,1	30,0	0,0	17,2	48,7
K47A	3,9	31,2	0,0	18,1	46,8

Supplementary Data S4. Far-UV CD spectrum (mean residue ellipticity, MRE) of CCL26 and ΔP53-L71. ΔP53-L71 lacks the amino acids associated with the α-helix. Using circular dichroism it could be confirmed that the typical α-helical structure is missing in the truncation mutant

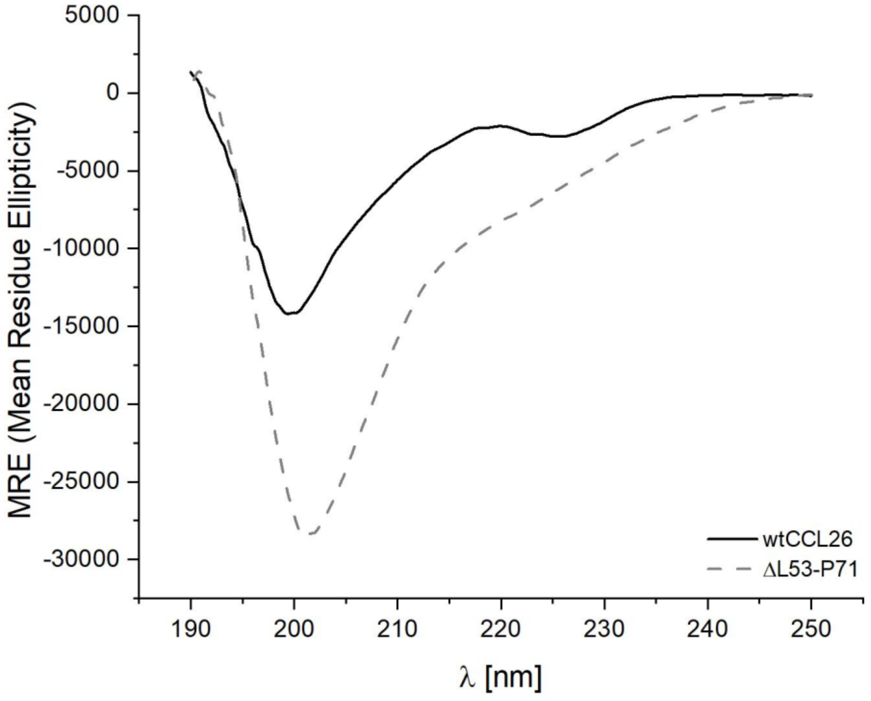

Supplementary Data S5. Guanidine-induced unfolding (GdmCl) curves of wtCCL26 and its mutants, Denaturation was followed by bisANS coupling leading to a change in emission intensity at a given GdmCl concentration. Data was analyzed using the Boltzmann equation, calculating the unfolding transition GdmCl midpoint concentration (x0) and the relative cooperativity

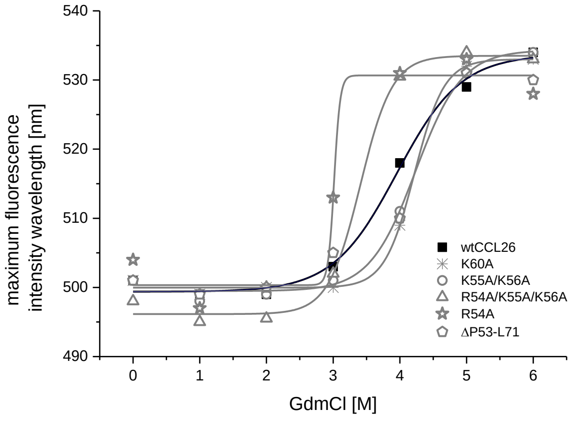

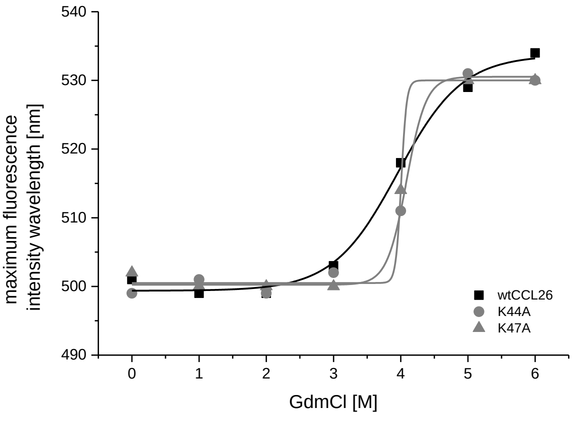

Supplementary Data S6. Guanidine-induced unfolding (GdmCl) of wtCCL26 and its mutants. The Calculated GdmCl midpoint concentration (x0) and the relative cooperativity (narrower range of GdmCl concentration required for unfolding) is comparable for all porteins and the unfold between 3 an 4,2 M and are thus quite stable

Protein	X0 (nM)	dx
wtCCL26	4,0 ± 0,09	0,48
CCL26 K60A	4,2 ± 0,07	0,22
K55A/K56A	4,2 ± 0,08	0,35
R54A/K55A/K56A	3,4 ±-0,08	0,24
R54A	3,0 ±-0,02	0,05
ΔP53-L71	4,2 ± 0,32	0,19
K44A	4,1 ± 0,46	0,15
K47A	4,0 ± 0,01	0,04
